# A Preliminary Study on the Use of Highly Aromatic Pyrolysis Oils Coming from Plastic Waste as Alternative Liquid Fuels

**DOI:** 10.3390/ma16186306

**Published:** 2023-09-20

**Authors:** Asier Asueta, Laura Fulgencio-Medrano, Rafael Miguel-Fernández, Jon Leivar, Izotz Amundarain, Ana Iruskieta, Sixto Arnaiz, Jose Ignacio Gutiérrez-Ortiz, Alexander Lopez-Urionabarrenechea

**Affiliations:** 1 GAIKER Technology Centre, Basque Research and Technology Alliance (BRTA), Parque Tecnológico de Bizkaia, Edificio 202, 48170 Zamudio, Spain; asueta@gaiker.es (A.A.);; 2 Chemical Engineering Department, Faculty of Science and Technology, University of the Basque Country (UPV/EHU), 48940 Leioa, Spain; 3 Chemical and Environmental Engineering Department, Faculty of Engineering of Bilbao, University of the Basque Country (UPV/EHU), Plaza Ingeniero Torres Quevedo 1, 48013 Bilbao, Spain

**Keywords:** pyrolysis, WEEE plastics, feedstock recycling, plastic waste, thermal decomposition, alternative fuels, pyrolysis oils, factorial design

## Abstract

In this work, the low-temperature pyrolysis of a real plastic mixture sample collected at a WEEE-authorised recycling facility has been investigated. The sample was pyrolysed in a batch reactor in different temperature and residence time conditions and auto-generated pressure by following a factorial design, with the objective of maximising the liquid (oil) fraction. Furthermore, the main polymers constituting the real sample were also pyrolysed in order to understand their role in the generation of oil. The pyrolysis oils were characterised and compared with commercial fuel oil number 6. The results showed that in comparison to commercial fuel oil, pyrolysis oils coming from WEEE plastic waste had similar heating values, were lighter and less viscous and presented similar toxicity profiles in fumes of combustion.

## 1. Introduction

The large increase in the generation of plastic waste, due to the great success of these materials for numerous daily applications, has caused growing interest in eco-efficient strategies for its appropriate management. The global concern about compliance with the principles of sustainable development imposes severe penalties on traditional waste management procedures based on disposal and destruction by incineration without any resource recovery. Therefore, the improvement of technologies for the treatment and recycling of problematic plastic waste is crucial. In this scenario, chemical recycling of plastic waste is currently a hot topic in Europe [1,2]. Different government policies show that administrations seem to have finally realised that this is a real and technically feasible alternative to treat many plastic waste products that, due to technical and/or economic reasons, are left out of the mechanical recycling route.

Pyrolysis is one of the techniques considered to be a chemical recycling alternative. It is a thermal process (350–900 °C) in the absence of fed air and it can produce solid, gaseous and liquid products of different characteristics and industrial value depending on the type of waste and the operating variables [3]. When post-consumer plastic waste is pyrolysed, the desired product is usually liquid, which is commonly known as pyrolysis oil because it is a mixture of organic substances in the same way as mineral oils or refinery streams. The focus of interest is currently on producing pyrolysis oils that can partially replace the naphtha that feeds the steam cracker in petrochemical processes [4,5]. This is because the olefins produced in this unit are used to make polyolefins such as polyethylene or polypropylene, so using pyrolysis oil from plastic waste would mean that these final polyolefins would have a certain percentage of recycled content. This is of utmost importance today because plastics made in this way are very likely to be “appropriate technology” according to the new European regulation on recycled plastic materials for use in contact with food [6]. This may be the only way for some materials to ensure the recycled content required by the new European directive for the reduction in the impact of certain plastic products on the environment [7] since mechanically recycled plastic is only allowed for PET in this application. The point is that the aromatic content of naphtha is usually lower than 30 wt.% and ideally lower than 20 wt.% [8]. These compositional requirements are far different from those of pyrolysis oils coming from some kind of plastic waste, e.g., oils produced in the pyrolysis of the plastic fraction of waste electrical and electronic equipment (WEEE). WEEE plastics are very rich in styrenic polymers such as polystyrene (PS), acrylonitrile butadiene styrene (ABS), styrene acrylonitrile (SAN), acrylonitrile styrene acrylate (ASA), or styrene butadiene (SB), which produce very aromatic oils in pyrolysis conditions, preventing their use as a naphtha substitute [9,10].

The only possible use of these aromatic oils as secondary raw materials in petrochemical processes would be as feed to the aromatic fractionation processes for the production of benzene, toluene and xylenes (BTX), but its complex composition makes it an unattractive option at present [11]. Therefore, one alternative for the industrial exploitation of these aromatic oils could be their use as alternative liquid fuels in industrial boilers or kilns [12,13]. This option, although it is an alternative of lower value than recycling according to the European Union waste hierarchy, can be important and competitive with the “plastic to plastic” route in some specific cases in terms of life cycle assessment, as the work of Das et al. reveals [14].

In this work, the production of pyrolysis oils from real plastic waste collected at a WEEE-authorised recycling facility will be presented. The maximisation of the liquid fraction in the pyrolysis process will be studied by means of the theory of experiments based on 2^k^ factorial design and the oil coming from the optimised process will be compared with commercial fuel oil number 6 [15]. Pyrolysis of WEEE plastics is a subject that has been studied for many years [16,17]. Published work has focused mainly on the role of halogens and dehalogenation processes [18,19,20] and on the use of catalysts to improve the properties of pyrolysis products [21,22,23]. All these studies indicate that pyrolysis liquids can be used as alternative fuels due to their high calorific value, but none of them make comparisons with traditional fuels. Therefore, the novelty of this work lies in the comparison of the properties of WEEE pyrolysis oils with commercial fuel, especially in terms of combustion emissions. In this sense, the authors think that progress needs to be made in these types of studies if it is intended to advance the circularity of waste and the use of products derived from them.

## 2. Materials and Methods

### 2.1. Materials to Be Pyrolyzed

Two types of materials were used in this study. On the one hand, a set of virgin plastics were used, which are usually part of electric and electronic equipment: Polypropylene (PP), high-density polyethylene (HDPE), polystyrene (PS), acrylonitrile butadiene styrene (ABS), acrylonitrile styrene acrylate (ASA), styrene acrylonitrile (SAN) and styrene butadiene (SB). These plastics were provided by Spanish chemical companies in pellet size (~3 mm). On the other hand, a real waste sample collected from the rejects of a local WEEE recycling facility was also used. In this industrial installation, after the separation of hazardous elements and big plastic parts, the rest of the materials comprising WEEE are separated by mechanical operations (shredding and sorting) in different streams in order to mainly recover metals (steel, aluminium and copper). Plastics that are not separated in the first step are therefore concentrated in streams that usually constitute the plant’s rejects, as they are difficult to valorise. The difficulty lies in the fact that these streams are composed of many plastic materials with a very small particle size (they come from milling processes), which also include other materials that have not been adequately sorted in the process (metals, glass, rubbers, or wood). Taking into account the cost and difficulty of the material separation from these rejected streams and the incompatibility between the different types of polymers when mixed for mechanical recycling, currently, the only economically feasible management option is incineration or a landfill. The sample for this study was representatively obtained from such rejects in order to determine if a recycling alternative was possible. This sample, called the WEEE sample, was ground to 5 mm particles prior to use.

### 2.2. Pyrolysis Experiments

The pyrolysis experiments were carried out in a batch unstirred tank reactor of 1.8 dm^3^ volume, made of stainless steel and able to operate up to 250 bar and 500 °C. The reaction system included automated control, real-time parameter monitoring, gas feeding lines and manual sampling for liquids, vapours and gases. In a typical run, 50 g of the material to be tested is placed into the reactor, which is sealed. Then nitrogen is passed through at a rate of 500 cm^3^ min^−1^ to remove air and the system is closed and heated up at a rate of 15 °C min^−1^ to the reaction temperature (T), which is maintained during a specific period of time (residence time, t_r_). The process occurs under auto-generated pressure. When finished, the reactor is cooled down and the uncondensed gas fraction is collected in Tedlar^®^ plastic bags. Liquid and solid pyrolysis yields are determined by weighing the amount of each fraction obtained (once they are separated by filtration) and calculating the corresponding percentage as shown In Equations (1) and (2), while the gaseous fraction yield is determined by difference.

(1) $$Liquid\;yield\;(\%)=\frac{W_{\;liquid\;product}}{W_{pyrolysed\;sample}}\cdot 100$$

(2) $$Solid\;yield\;(\%)=\frac{W_{solid\;residue}}{W_{pyrolysed\;sample}}\cdot 100$$

**Figure 1:**
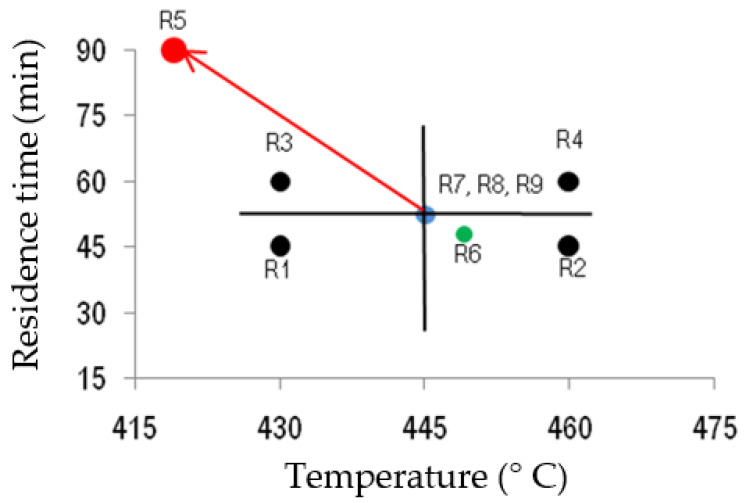
Experimental points (R=run) considered in the design of experiments, where 430 is x_1_ = −1, 445 is x_1_ = 0, 460 is x_1_ = +1, 45 is x_2_ = −1, 53 is x_2_ = 0 and 60 is x_2_ = +1.

Determining the optimum operating conditions to maximise the production of pyrolysis oils from the real waste sample was one of the issues to be investigated. For this purpose, an approach of the theory of experiments based on 2^k^ factorial design was developed, identifying temperature as the x_1_ variable, residence time as the x_2_ variable and the liquid yield as the response variable [24]. The experimental domain of x_1_ was 430–460 °C while 45–60 min was that of x_2_, and both of them were chosen due to previous studies and experience of the research group and based on the thermogravimetric profile of the real sample. The factorial design was based on the generation of a reasonable point distribution in the interest region with the lower number of experiments. At the same time, the objective was to ensure that for each point, the adjusted value is as close as possible to the real value. The initial adjustment followed a first-order model, then the maximum slope criterion was applied by searching for areas of the experimental region where a significant increase in the liquid yield was expected and, finally, an adjusted second-order model was obtained. The experimental error was estimated by means of repetition of some observations in the centre of the experimental design and calculated following Equation (3). So, the design of experiments included the following runs: 4 experiments related to 2^2^ factorial designs (Runs #1 to #4, 430/45, 460/45, 460/60, 430/60, respectively), 1 experiment following the maximum slope criterion (Run #5, 420/90), a proposed experiment to view the effect of moving away from the centre (Run #6, 450/48) and 3 repetitions in the centre of the design (Run #7–#9, 445/53). The graphical representation of the experimental conditions used is shown in Figure 1.

(3) $$\sigma^{2}=\frac{\sum_{R7}^{R9}{\left(Liquid\;yield\right)}^{2}-{\left({\bar{Liquid\;yield}}_{R7,\;R8,\;R9}\right)}^{2}}{2}\cdot 100$$

### 2.3. Analytical Techniques

The WEEE sample was characterised using the following analytical techniques. The identification of materials was carried out using KUSTA 4004M equipment based on near-infrared spectroscopy (LLA Instruments GmbH, Berlin, Germany) and supported by fire behaviour tests for plastics. The elemental composition was determined with the automatic analysers LECO CHN-2000 and CNS-2000 (St. Joseph, MI, USA) over dry matter. The higher heating value (HHV) was measured in the automatic calorimeter 1356 Parr Instrument (St. Moline, IL, USA), following the EN ISO 18125:2018 standard. The ash content was determined by means of calcination in air following the European standard method EN ISO 21656:2021. The halogen content was determined by combustion in the above-mentioned calorimeter followed by high-performance liquid chromatography (HPLC) of the halides-containing basic solution (KOH, 0.2 M) in the ion chromatograph DIONEX ICS-1000 (Sunnyvale, CA, USA) as stated in the European standard EN 14582:2016. The metals in the sample were detected and quantified through inductively coupled plasma atomic emission spectroscopy (ICP/AES, Perkin Elmer OPTIMA 2100DV Optical, Waltham, MA, USA), following the method 6010 of the US EPA, after a previous acid digestion defined in the EPA 3052 method. For measuring the apparent density, the ASTM D1895 standard was used. The thermogravimetric behaviour of the WEEE sample was studied using a TA-Instruments TGA/Q5000 analyser (New Castle, DE, USA). The analysis was conducted by heating 5 mg of an additionally finely ground WEEE subsample (<1 mm) under nitrogen flow (50 mL min^−1^) to 900 °C at a rate of 5 °C min^−1^.

Regarding pyrolysis liquids and commercial fuel oil, their composition was analysed by gas chromatography/mass spectrometry (GC/MS, Agilent 6890, Santa Clara, CA, USA) for chemical identification and by gas chromatography coupled with a flame ionization detector (GC/FID, Agilent 6890, Santa Clara, CA, USA) for carbon atom number determination. Hexane (C6), dodecane (C12), hexadecane (C16) and triacontane (C30) were used as standards. HHV and halogen content were determined by the same procedures explained above for the WEEE sample. The solid content was established by means of filtration using a 0.45 µm pore size Millipore (Burlington, MA, USA) membrane (Millex filter). Furthermore, the density and viscosity of the liquids were determined using a pycnometer and a Brookfield LVDVII viscometer (Middleboro, MA, USA), respectively. Finally, the toxicity of the fumes derived from the combustion of the pyrolysis oil and the commercial fuel oil was evaluated through the AFNOR NF X70-100-1 (2006) steady-state tube furnace methodology for the determination of hazardous components of fire effluents (Part 1: Methods for analysing gases stemming from thermal degradation and Part 2: Tubular furnace thermal degradation method). The test method consists of burning a known amount of sample in a tubular furnace with a synthetic airflow at a temperature to be determined, up to 900 °C, for 20 min. In this case, tests were carried out in a Lenton LTF 12/75/610 tubular furnace (Hope Valley, UK) at 600 °C according to Section 6.3.1 in the NF F16-101 (1988) standard with approximately 1 g of sample. The gases generated in the combustion are dragged by the airflow and later collected in solution or in a gaseous state, depending on the analytical technique used for detection and quantification. Analytical methods used to quantify each gas were those recommended in AFNOR NF X70-100-1 standard, specifically the following ones. (1) Non-dispersive infrared spectroscopy (Siemens Ultramat 23, Munich, Germany) for CO and CO_2_ (Sections 7.1.1 and 7.1.2 of NF X70-100-1 standard, respectively). (2) Ionometry with selective electrodes (Mettler Toledo perfectIONTM DX series, Schwerzenbach, Switzerland) for HF (Section 7.2.2 of NF X70-100-1 standard). (3) Ion liquid chromatography (DIONEX ICS-1000 (Sunnyvale, CA, USA) for HCl, HBr and SO_2_ (Sections 7.3.2, 7.4.2 y 7.6 of NF X70-100-1 standard, respectively. (4) Spectrophotometry (Shimadzu UV-1800, Kyoto, Japan) for HCN (Section 7.5.1 of NF X70-100-1 standard). CO, CO_2_ and HCN were used as asphyxia-producing substances and HF, HCl, HBr and SO_2_ as chemical species giving rise to irritant effects.

## 3. Results and Discussion

### 3.1. WEEE Sample Characterization

**Figure 2:**
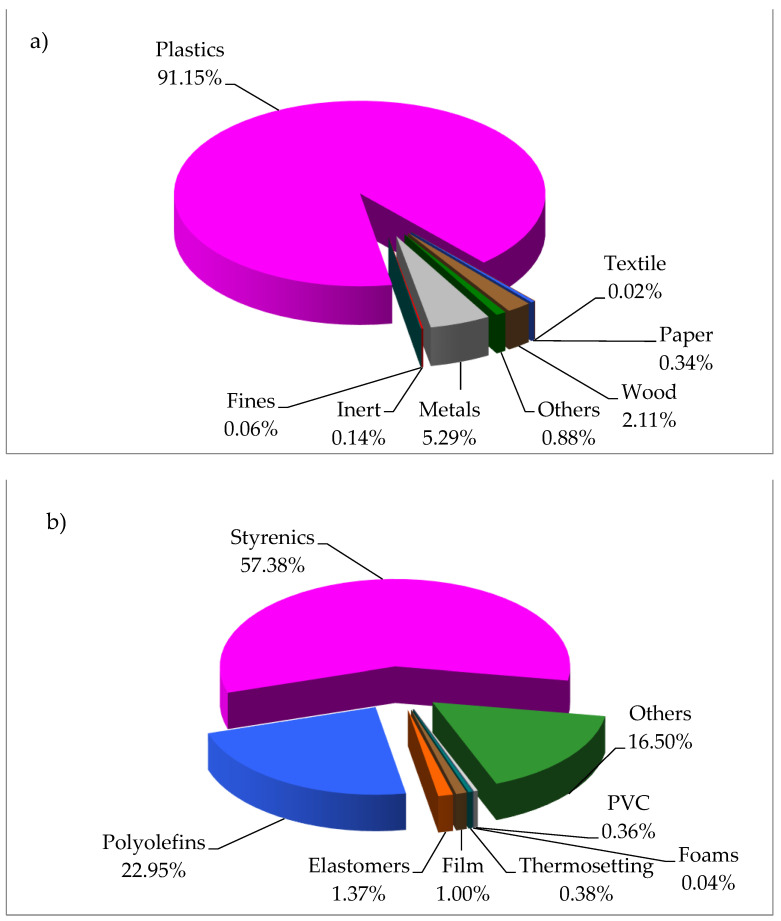
Material composition of WEEE sample. (**a**) Total composition; (**b**) composition of plastics.

Figure 2 shows the material composition of the WEEE sample. As can be seen, this sample was mainly a mixture of plastics (91.2 wt.%), metals (5.3 wt.%), wood (2.1 wt.%) and other materials (1.4 wt.%). In the plastic fraction, styrenics and polyolefins were the predominant families of polymers (52.3 wt.% and 20.9 wt.%, respectively). The styrenic fraction was mainly composed of PS, but co-polymers such as ABS, SAN and SB were also present. In the case of polyolefins, polypropylene (PP) was by far the main plastic (only traces of polyethylene, PE, were found). Then, 15 wt.% of other plastics were also detected, specifically 5.3 wt.% of polyamide (PA), 3.9 wt.% of polycarbonate (PC), 2.7 wt.% of poly(methyl methacrylate) (PMMA), 1.5 wt.% of polyoxymethylene (POM), 0.8 wt.% of polyethylene terephthalate (PET) and 0.8 wt.% of polybutylene terephthalate (PBT). The rest of the plastics were rubbers, films, thermosets, foams and poly(vinyl chloride) (PVC). The composition of this sample is very similar to other real WEEE plastic samples used by authors in previous research works [10,17]. However, the PVC content (0.3 wt.%) was very low in comparison with those samples, which is very good taking into account that the presence of chlorine in oils is quite problematic due to corrosion and environmental issues [12,13].

Table 1 shows the organic and inorganic elemental composition of the WEEE sample, which includes CHNS and halogens in the fuel side of the waste (organic) and the metals present in the ash (inorganic). First of all, it can be seen that more than 80 wt.% of the sample was organic, from which the pyrolysis oils must be obtained, so the competitiveness of the pyrolysis process was ensured in this case. As expected, carbon was the main organic element, followed by hydrogen and nitrogen. Then, small quantities of halogens, especially chlorine, were also detected. The chlorine content is higher than expected in view of the low percentage of PVC in the sample. However, chlorine is commonly found in plastics other than PVC and even more likely in the case of WEEE plastics, where it may be part of additives such as flame retardants. This is likely also the origin of the detected bromine. Finally, sulphur and fluorine showed amounts below the detection limit of the analytical techniques used. The results of ash content and organic elemental analysis are similar to those obtained for this type of sample in previous works carried out by the authors of this article and by other research groups [10,25,26,27].

With regards to the inorganic content of the sample, it should be noted that the ash content (16.2 wt.%) was higher than the content of inorganic materials found in the material characterisation of the sample (metals + inert material + fines (mostly) ≈ 5.5 wt.%). This indicates that there was likely a significant quantity of inorganic fillers/additives in the plastic fraction of the sample. Concerning the metal content, Table 1 shows that the more abundant metals in the WEEE sample were Cu, Ca, Al, Sb, P, Sn and Zn. Cu, Al, Sn and Zn were mainly associated with the metal fraction of this sample (see Figure 2), although they may also be present as fillers or additives. However, this is more typical for metals such as Ca, Sb and P. Copper, aluminium and calcium are very characteristic metals of these plastic WEEE fractions and have been previously reported as the main metals present in the ashes of this type of waste [10,26]. In this case, high concentrations of antimony were also obtained, likely indicating the presence of antimony trioxide as a flame retardant in some of the plastics in this sample [28]. Apart from the data shown in Table 1, complementary physicochemical parameters were determined in the sample: HHV was 33.6 ± 0.4 MJ kg^−1^ and the apparent density was 496 ± 26 kg m^−3^.

**Figure 3:**
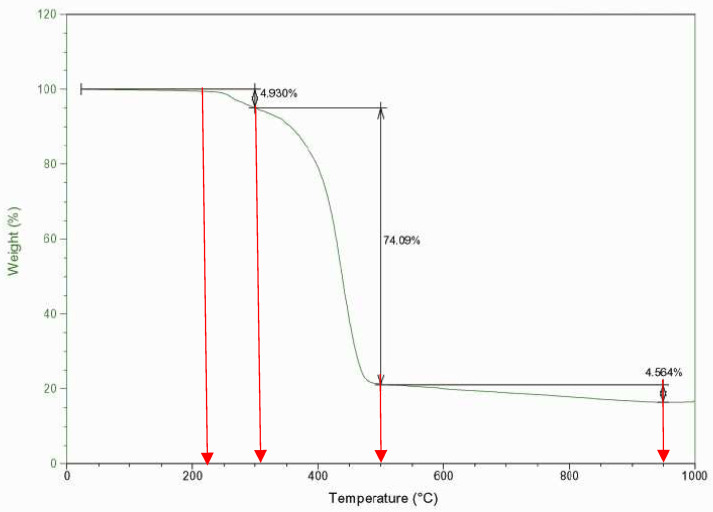
Thermogravimetric profile of WEEE sample.

Thermogravimetric analysis was carried out in order to evaluate the thermal behaviour of the WEEE plastic sample. Figure 3 shows the thermogravimetric profile of the sample, including the weight loss of the main decomposition steps. It can be seen that sample thermal degradation comprised three different weight loss steps. The main degradation phenomenon of the sample took place at temperatures in the range 300–500 °C and then another two and less important degradation steps occurred between 200 and 300 °C and 500 and 950 °C. These decomposition phenomena can be well related to the material composition of the sample. The first weight loss, which occurred at the lowest temperatures, was likely mainly related to dehalogenation processes, since at temperatures between 250 and 350 °C, the carbon-halogen bonds break, with the characteristic temperature around 300 °C [10,25,29]. In this case, this characteristic temperature seems to show a lower value, but it is possible that it was influenced by some other decomposition processes that occur at low temperatures, such as the decomposition of wood hemicellulose, the dissociation at unsaturated end chains of PMMA or the dehydration reactions of some thermosets, for example, those based on polyester [30]. The weight loss of this step is greater than the PVC content of the sample, but it should be noted that any dehalogenation processes resulting from halogenated additives (flame retardants, fillers, etc.) that may be present in other plastics also occur at these temperatures. Moreover, Esposito et al. also suggested that the weight loss of a WEEE plastic sample at around 250 °C could be related to the initial decomposition of epoxy or phenolic thermosets since they detected formaldehyde by FTIR analysis at such temperatures [26].

The second and most important weight loss (300–500 °C) is related to the two main plastic families of the sample: Styrenic plastics and polyolefins. PS is a commodity plastic that starts decomposition at temperatures lower than 400 °C since polyolefins, PP and PE, quantitatively decompose slightly before 500 °C [31]. Finally, a slight constant weight loss can be observed between 500 and 950 °C. This may be related, on the one hand, to the presence of calcium carbonate, a very common filler in plastic formulations that decomposes in the 600–800 °C range [30]. Above these temperatures, weight losses are likely due to the loss of volatiles from the char formed in the previous decomposition stages. This thermogravimetric behaviour defined the limits of the temperature domain for the design of experiments. The aim was to find a temperature that ensured that the main decomposition phenomenon occurred quantitatively (step 2) and, at the same time, was not too high so as not to favour the production of gas at the expense of the liquid. Taking into account that the thermal inertia of reactors is greater than that of thermogravimetric analysers and not wishing to exceed 500 °C in any case, a range of 430–460 °C was chosen.

### 3.2. Pyrolysis Experiments

A set of reactions with the WEEE plastic sample were carried out with the aim of maximising the liquid yield. The results of the proposed experiments are shown in Table 2. As can be seen, the highest liquid yield (36.8 wt.%) was obtained at the central point of the experimental design (445 °C and 53 min). Any different conditions concerning temperature and time produced a decrease in the liquid yield. However, the reasons that explain this behaviour are quite likely to be different. On the one hand, in the test at the minimum temperature (420 °C, run #5), it was quite clear that the sample was not fully decomposed despite the long residence time used (90 min). This can be deduced from the high solid yield (46.4 wt.%) of this experiment. This result shows that temperature is a more important variable than residence time in batch tank reactors. This conclusion is also generally valid for pyrolysis processes regardless of the type of reactor used [3,31]. In other words, if a certain temperature is not reached, no matter how much residence time is used, it will not produce cracking of the sample. In this case, it seems that the key temperature was 430 °C since, from this temperature upwards, the solid yield stabilised at a value of around 30 wt.% and remained constant in the intervals of 430–460 °C and 45–60 min. It should be noted that the solid yield is higher than the ash content of the sample (16.2 wt.%), which indicates that some organic material remained in the ash after the pyrolysis experiments. This was the material commonly known as “char”, a carbonaceous substance resulting from the pyrolysis of some polymers, mainly those containing aromatic rings and cyclic structures with heteroatoms like nitrogen, oxygen or sulphur [32].

In the experiments carried out in the interval of 430–460 °C, the variations in the yields were not large enough to clearly define the effect of the operating variables since a variation of 5 points in yield at the same operating conditions could be, to some extent, acceptable when working with real samples. In these cases, the issues of heterogeneity do not normally allow one to ensure the representativeness of the samples in each test. As an example, run #1 seems totally anomalous, with a higher-than-expected gas production in view of the rest of the results. These types of unexpected deviations commonly occur in real waste pyrolysis processes and are due to the fundamental heterogeneity of the samples. Therefore, they must be taken into account when closing the mass balances of industrial processes, and here lies the importance of working with real waste samples at the research level.

**Figure 4:**
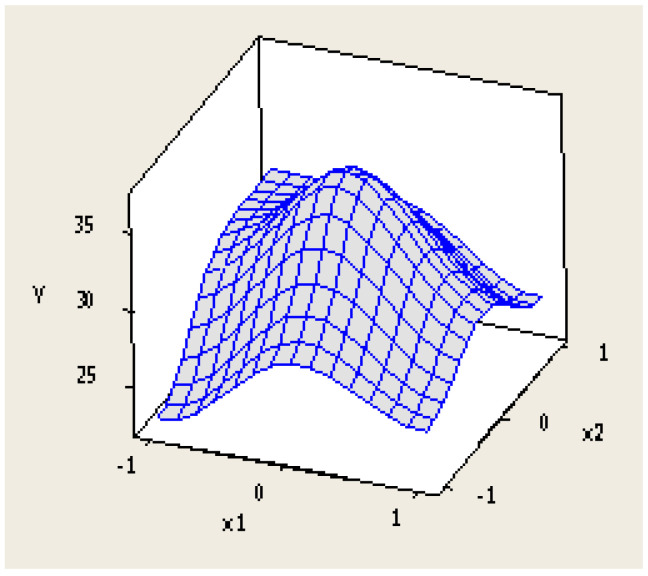
Three-dimensional response surface.

Concerning the statistical analysis of the data obtained from the experimental design, the experimental error was calculated according to Equation (3). The result is shown in Equation (4). This experimental error can be attributed to the heterogeneity of the sample since there was very low variability among the results of the duplicated experiments and the applied methodology was identical. On the other hand, the three-dimensional response surface is shown in Figure 4. It can be observed that there is a significant curvature in the centre of the domain, which prevents the use of a first-order model. Consequently, the experimental results were adjusted to a second-order model, which is shown in Equation (5).

(4) $$\sigma^{2}=\frac{{\left(35.13\right)}^{2}+{\left(36.02\right)}^{2}\;{\left(36.81\right)}^{2}-{\left(107.96\right)}^{2}/3}{2}=0.706$$

(5) $$y=35.1718-0.77x_{1}+2.11x_{2}-1.85x_{1}\cdot x_{2}-9.20x_{1}{}^{2}-0.4x_{2}{}^{2}$$

In any case, it does seem that in the experiments at the highest temperature (460 °C), there was a greater generation of gases than in the rest (42–44 wt.%), except for the previously mentioned anomalous run #1. The increase in the production of gases at the expense of liquids with increasing temperatures is a known fact in the pyrolysis of plastic waste, which supports this appreciation [3]. Therefore, by discarding this temperature and 420 °C due to insufficient decomposition of the sample, it can be concluded that the optimum temperature range for the production of oil from this sample is 430–450 °C, with residence times between 45 and 60 min.

In addition to the experiments with the real sample, experiments with the main types of polymers that were part of the real sample were also carried out in this work. The aim was to draw conclusions regarding which type of polymers generated the highest amounts of pyrolysis oil. This would allow a hypothetical industrial pyrolysis plant receiving such samples of WEEE plastics to select materials with the aim of maximising the liquid product. The solid, liquid and gas yields (wt.%) obtained in the pyrolysis experiments carried out with the selected materials are presented in Table 3. These experiments were carried out at operating conditions within the optimal working range defined with the real sample, namely 430 °C and 60 min. Table 3 shows that the pyrolysis of PP, HDPE and PS maximised the production of liquids, yielding around 80 wt.% of oils or even more in the case of PS. In contrast, the styrenic co-polymers (ABS, ASA, SAN and SB) produced much lower liquid yields (45–68 wt.%), mainly due to the generation of a significant proportion of solid material (char). Therefore, from this series of experiments, it can be concluded that styrenic co-polymers penalise the production of oils in the pyrolysis processes of WEEE plastics, also generating a solid fraction that is non-existent when strictly pyrolysing PP, HDPE and PS. Taking into account that the sum of PS, PP and HDPE constituted almost 60 wt.% of the real sample of WEEE plastics, a sorting process prior to the pyrolysis process to remove the styrenic co-polymers together with the rest of the non-polymeric materials seems to be a reasonable option.

Table 3 also shows some experiments in which mixtures of materials were used. The aim of these tests was to detect possible interactions between the products derived from the pyrolysis of each material, which could both promote or penalise oil production. The main conclusion reached was that no relevant effect of the studied interactions (PP/HDPE and ASB/SB) was observed. In all cases, the experimental liquid yield did not differ by more than 4 points from the theoretical yield, which can be calculated from the yields of the experiments with the individual materials.

### 3.3. Pyrolysis Oils

Table 4 shows the composition of the pyrolysis oils obtained from the real sample of WEEE plastics. Specifically, and by way of example, the main substances found in the liquids of run #7 are shown. As can be seen, 70% area of the sample was made up of aromatic hydrocarbons, phenol and its derivatives, which demonstrates the aromatic nature of these liquids. This result agrees with those obtained by the authors working with similar waste samples [10,19]. The composition of the oils also suggests that the use of these liquids as a feedstock for BTX fractionation processes is complicated. On the one hand, the proportion of BTX did not seem to be very relevant in these liquids (they were not among the main components). On the other hand, the presence of phenol and its derivatives suggests that these liquids had a higher oxygen content than that allowed in the common feed of this petrochemical process, where low-oxygen aromatised naphtha is normally used [33].

Consequently, the possibilities of these oils as alternative fuels must be evaluated. For this, Table 5 shows the HHV and carbon atom distribution of the liquids obtained in the pyrolysis of the real sample in comparison with the same properties of fuel oil number 6. It is clear that the heating value of the pyrolysis oils is high enough to be used as a fuel since all the liquids ranged around 40 MJ kg^−1^, while that of the commercial fuel oil was 45 MJ kg^−1^. On the other hand, there were significant differences in the carbon atom distribution between pyrolysis oils and commercial fuel oil. In general terms, pyrolysis oils were very much lighter than fuel oil 6. The formers were mainly composed of substances in the range of C_7_–C_12_ while the latter presented the maximum value for C_17_–C_30_ substances. This was, in some way, a good result since it was found that pyrolysis oils did not constitute a fuel as heavy as fuel oil 6, also known as heavy or residual fuel oil.

As in the case of oil yield, the influence of each of the main polymers in the sample on the two properties shown in Table 5 (HHV and carbon atom distribution) was also studied. The results are shown in Table 6. In this case, the main differences appeared between the two large families of polymers: Polyolefins and styrenics. On the one hand, polyolefins maximised the HHV of liquids but presented a distribution of carbon atoms with a significant relative weight of heavy substances (C17–C30). In this sense, the pyrolysis oil from HDPE presented practically the same results as those of commercial fuel oil, both in HHV and in carbon atom distribution. On the other hand, styrenics were responsible for the decrease in HHV but also for the increase in the proportion of light substances. Therefore, in this case, it can be said that controlling the content of styrenic plastics may favour a situation where the calorific value of the oils is high enough while having a shorter chain carbon atom distribution than that of commercial fuel oil. Once again, no concluding remark was obtained from the experiments of mixed virgin polymers (PP/HDPE and ABS/SB).

At last, additional analyses were carried out to evaluate the fuel properties of the pyrolysis oils from WEEE plastics. These are summarised in Table 7, where commercial fuel oil 6 and pyrolysis oil coming from run #9 are compared. It can be seen that the density was similar for both fuels, but the WEEE pyrolysis oil was less viscous due to its lighter nature when compared to commercial fuel, as stated in the previous paragraph. This was an interesting result from the point of view of minimising the needs and requirements of transport and pumping. In fact, commercial fuel oils classified as numbers 5 and 6 are claimed to be heated for transfer operations. This was something that pyrolysis oil did not need because it remained in a liquid state in room-temperature conditions. Concerning halogen and solid content, both fuels presented halogen content below the quantification level of the analytical technique and the solid content of the WEEE plastics pyrolysis liquid was about 5 wt.%, while commercial fuel did not show any solids. Finally, a similar profile in terms of toxicity of the emissions derived from the burning of the liquids was obtained. This was a very important result since it meant that pyrolysis oil did not produce additional toxicity in comparison with commercial fuel oil, which opens the possibility of using these oils as alternative liquid fuels in safe conditions.

## 4. Conclusions

The main conclusions that can be drawn from this work are the following:

▪ The rejected streams that are produced in WEEE recycling facilities are plastic-rich mixtures, mainly constituted by styrenic polymers and polyolefins. Moreover, other materials such as metals or wood can be found.
▪ The optimum operational parameters for the maximisation of the liquid yield from WEEE plastics in the lab-scale pyrolysis plant used in this research are temperatures in the range of 430–450 °C and residence times in the range of 45–60 min. In such conditions, the oil yield is 30–35 wt.%.
▪ Polyolefins and polystyrene are the plastics that maximise oil production in the pyrolysis process, without the generation of a solid product. On the other hand, styrenic co-polymers produce lower quantities of oil and a significant proportion of char.
▪ The pyrolysis oils of WEEE plastics are lighter than commercial fuel oil 6 and present similar higher heating values (≈40 MJ kg^−1^). In this case, polyolefins maximise the heating value but also the presence of heavy substances. In contrast, styrenics reduce the heating value at the same time they generate light substances.
▪ In combustion conditions, the pyrolysis oils of WEEE show the same toxicity profile in fumes as that of the commercial fuel oil 6.
▪ Future research must be focused on determining specific properties of the pyrolysis oils that can limit their use as alternative fuels, and among others, the exact concentration of halogens or the solid formation after combustion. Another interesting area of investigation is the distillation of pyrolysis oils in order to obtain liquids with narrower carbon atom distribution, which are more easily comparable to liquid fossil fuels.

## Figures and Tables

**Table 1:** Organic and inorganic elemental composition of the sample.

Organic/Inorganic	Element	Value (wt.% for CHNS/ClBrF and mg/kg for Metals)
Organic content (83.8 wt.%)	C	75.0
	H	7.9
	N	1.5
	S	<0.02
	Cl	1.4
	Br	0.7
	F	<0.02
Ash content ^1^ (16.2 wt.%)	Al	2727
	Cu	37,068
	Zn	1135
	Ca	7891
	Sb	3537
	P	1304
	Sn	1689

^1^ Other metals detected below 1000 mg/kg were Cd 23.6, Cr 50.4, Ni 58.8, Pb 163.6, Fe 868, Mn 45.9, K 94.6, Mg 842, Mo 5.07, Hg 0.2, Si 196, Ag 14.5, As 7.24, Ba 35.5, Se 11.3, Co 10.6, Te 14.3. Other metals detected below the quantification limit were (mg/kg) V < 5, Na < 50, Rh < 10, Be < 5, Pd < 10, Pt < 10.

**Table 2:** Pyrolysis yields of WEEE sample (wt.%).

Run	T (°C)	t_r_ (min)	Liquid	Solid	Gas *
#1	430	45	22.6	28.4	49.0
#2	460	45	25.2	32.8	42.0
#3	460	60	24.8	31.2	44.0
#4	430	60	29.7	30.6	39.7
#5	420	90	25.4	46.4	28.2
#6	450	48	30.6	30.2	39.2
#7	445	53	35.1	32.9	32.0
#8	445	53	36.0	31.8	32.2
#9	445	53	36.8	31.2	32.0

* By difference.

**Table 3:** Pyrolysis yields of virgin plastics at T = 430 °C and t_r_ = 60 min (wt.%).

Sample	Nature	Liquid	Solid	Gas *
PP	Polyolefinic	81.0	0.0	19.0
HDPE		78.9	0.0	21.1
75 wt.% PP + 25 wt.% HDPE		78.0	0.0	22.0
50 wt.% PP + 50 wt.% HDPE		80.0	0.0	20.0
25 wt.% PP + 75 wt.% HDPE		83.0	0.0	17.0
PS	Styrenic	85.2	0.1	14.7
ABS		45.1	31.6	23.3
ASA		56.6	18.8	24.6
SAN		67.2	23.9	8.9
SB		68.5	19.1	12.4
60 wt.% ABS + 40 wt.% SB		50.4	20.7	28.9
40 wt.% ABS + 60 wt.% SB		59.8	17.1	23.1

* By difference.

**Table 4:** Main components of WEEE plastic pyrolysis liquids identified by GC/MS (% area).

Compound	Run #7
Ethylbenzene	30.0
Benzene, 1,3-dimethyl-	4.3
Benzene, (1-methylethyl)-	16.4
Benzene, propyl	4.3
Benzene, 1,3,5-trimethyl-	2.7
Phenol	3.2
Benzene, 1-methyl-4-propyl-	1.7
Phenol, 2-methyl-	1.9
Phenol, 4-(1-methylethyl)-	3.5
Naphthalene 2-methyl-	1.9
TOTAL IDENTIFIED	70.0
NON-IDENTIFIED	30.0

**Table 5:** HHV (MJ kg^−1^) and carbon number composition (GC/FID, % area) of pyrolysis oils of WEEE sample and No. 6 Fuel oil.

Experiment	HHV	C_7_–C_12_	C_13_–C_16_	C_17_–C_30_
#1	41.4	71.6	9.8	18.6
#2	40.3	79.4	7.4	13.2
#3	39.2	85.7	6.1	8.1
#4	40.7	79.6	7.1	13.2
#5	40.5	93.0	3.4	3.5
#6	40.9	82.8	7.0	10.2
#7	39.8	91.4	4.5	4.1
#8	40.8	90.4	4.7	4.9
#9	40.5	84.4	6.3	9.3
Fuel oil 6	45.0	35.9	26.7	37.3

**Table 6:** HHV (MJ kg^−1^) and carbon number composition (GC/FID, % area) of pyrolysis oils of virgin plastics.

Sample	HHV	C_7_–C_12_	C_13_–C_16_	C_17_–C_30_
PP	44.3	56.9	22.9	20.2
HDPE	46.1	32.4	27.4	40.2
75 wt.% PP + 25 wt.% HDPE	41.7	50.4	23.0	26.6
50 wt.% PP + 50 wt.% HDPE	43.3	50.3	23.2	25.6
25 wt.% PP + 75 wt.% HDPE	43.9	40.3	25.2	34.5
PS	40.2	76.1	7.8	16.1
ABS	38.7	79.4	10.1	10.5
ASA	37.4	74.0	12.2	13.7
SAN	38.2	63.2	15.1	21.7
60 wt.% ABS + 40 wt.% SB	39.8	63.0	14.5	22.5
40 wt.% ABS + 60 wt.% SB	40.5	9.9	19.7	40.5

**Table 7:** Comparison of fuel properties between WEEE plastic sample pyrolysis liquid and commercial fuel oil 6.

Property		WEEE Oil #9	Commercial No. 6 Fuel Oil
Density (kg m^−3^)		879	900
Viscosity (mm^2^ s^−1^)		1.31	2.0–4.5
Halogens (mg g^−1^)	Cl	<0.35	<0.35
	Br	<0.07	<0.07
	F	<0.07	<0.07
Solid content (wt.%)		5	-
Toxicity (mg g^−1^)	CO	48.2	48.6
	CO_2_	406.5	466.5
	HCl	<2.2	<2.2
	HBr	<1.1	<1.1
	SO_2_	<2.2	<2.2
	HF	<0.1	<0.1
	HCN	<1.1	2.0
